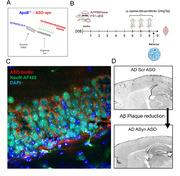# Downregulation of α‐synuclein in a mouse model of Alzheimer’s Disease: neuropathological, behavioral and transcriptomic changes

**DOI:** 10.1002/alz.092752

**Published:** 2025-01-03

**Authors:** Andre Direito Goulart Leitao, Rijwan Uddin Ahammad, Brian Spencer, Chengbiao Wu, Robert A. Rissman

**Affiliations:** ^1^ Department of Neurosciences, University of California San Diego, La Jolla, CA USA; ^2^ Department of Physiology and Neuroscience, Alzheimer’s Therapeutic Research Institute, Keck School of Medicine of the University of Southern California, San Diego, CA USA; ^3^ Alzheimer’s Therapeutic Research Institute, Keck School of Medicine, University of Southern California, San Diego, CA USA

## Abstract

**Background:**

Neurodegenerative disorders of aging are characterized by the progressive accumulation of proteins such as α‐synuclein (α‐syn) and amyloid beta (Aβ). Misfolded and aggregated α‐syn has been implicated in neurological disorders such as Parkinson’s disease (PD), and Dementia with Lewy Bodies (DLB), but less so in Alzheimer’s Disease (AD) despite the fact that synuclein pathology is present in over 50% of postmortem brains of AD patients. We are now expanding on our previous studies which showed positive therapeutic effects of downregulating α‐syn in AD mice to understand the overall brain transcriptomic and mechanistic changes induced by treatment.

**Method:**

We first treated AD mice systemically with a α‐syn antisense oligonucleotide (ASO‐syn) conjugated with a LDLR‐specific peptide (ApoB^11^) that allows for transport across the blood‐brain barrier and measured the presence of the ASO in the brain by immunohistochemistry. We then treated both neurons in culture and 6 months‐old AD mice with the ApoB:ASO conjugates and measured α‐syn levels in both cells and brains by immunohistochemistry and Western blot to test the feasibility of this treatment to downregulate α‐syn. Finally, we performed single‐cell RNAseq to ask the question of how α‐syn interferes with neuropathology in AD, i.e. which genomic pathways are changed when mice are treated with ApoB:ASO‐syn conjugates.

**Result:**

We found that treatment of APP transgenic (AD) mice with ApoB^11^:ASO‐syn leads to a significant reduction of Aβ plaques burden, rescued neuronal loss and prevented astrogliosis, as compared to untreated AD mice. Importantly, we found that AD mice treated with the ASO‐syn had significantly improved spatial memory function. We found specific single‐cell transcriptomic changes associated with this downregulation of α‐syn, highlighting possible pathways for α‐syn‐mediated vulnerability.

**Conclusion:**

Collectively, our data supports the use of ApoB^11^:ASO α‐syn conjugates delivered systemically to downregulate α‐syn as a promising future therapeutic strategy in AD. Our work suggests that knocking down α‐syn could be necessary to prevent neuropathology in AD.